# Homelessness in early adulthood and biomedical risk factors by middle-age: the 1970 British Cohort Study

**DOI:** 10.1136/jech-2021-217457

**Published:** 2021-09-28

**Authors:** James W White, Mark Hamer, G David Batty

**Affiliations:** 1 Centre for Trials Research, Cardiff University, School of Medicine, Cardiff, UK; 2 Institute of Sport Exercise and Health, Division of Surgery and Interventional Science, University College London, London, UK; 3 Department of Epidemiology and Public Health, University College London, London, UK

**Keywords:** homeless persons, cardiovascular diseases, cohort studies

## Abstract

**Background:**

Homelessness has been linked to premature mortality but the relationship with biomedical risk factors is uncertain.

**Methods:**

We analysed data from 8581 participants in the 1970 British Birth Cohort Study. Homelessness and type of experience were self-reported at 30 years of age. Nine biomarkers outcomes were collected at 46 years of age: body mass index (BMI), blood pressure, total and high-density cholesterol, triglycerides, glycated haemoglobin, C-reactive protein (CRP), insulin-like growth factor 1 and we computed the 10-year risk for coronary heart disease.

**Results:**

By 30 years of age, 5.8% of participants had been homeless with sofa surfing the most common experience (4.3%). Homelessness was associated with socioeconomic disadvantage, mental health problems and substance use in early adulthood, but these differences were not expressed in biomarkers. After accounting for early adulthood characteristics, residing in a bed and breakfast was associated with a higher BMI (0.59, 95% CI 0.13 to 1.05) and CRP (0.16, 95% CI 0.04 to 0.29), squatting with a lower BMI (−1.69, 95% CI –3.08 to −0.21) and rough sleeping with a higher 10-year risk of coronary heart disease (0.03, 95% CI 0.01 to 0.05).

**Conclusions:**

Exposure to homelessness in early adulthood was essentially unrelated to biomarkers in middle age. Inconsistent links were found for specific types of experience.

## Introduction

Prospective and retrospective cohort studies have repeatedly shown that socioeconomic disadvantage in early life is associated with premature mortality.^1^ Such adversity has recently been more broadly defined to comprise not only economic hardship but also physical and emotional abuse, parental death and homelessness.^2^ Of these, exposure to homelessness is receiving increasing research attention. Although the literature is largely confined to data from high-income countries, rough sleepers and those attending homeless shelters have been found to have elevated mortality rates relative to the general population.^3^ Homeless status is, however, time-varying such that the majority of homeless people eventually become housed.^4^ Whether health risks are embodied in people who have been exposed to homeless but subsequently housed is unknown. We hypothesised associations between exposure to homelessness with increased biomedical risk factors implicated in chronic stress pathways, including lipids, inflammation and glucose metabolism.^5^

## Methods

We used data from the 1970 British Birth Cohort Study, an ongoing longitudinal study of children born in Great Britain between the 5 April 1970 and 11 April 1970. A total of 16 571 babies born in England, Scotland and Wales were enrolled at birth and have been followed-up on 10 occasions across the life course. For the purposes of the present analyses, we used data on experiences of homelessness recorded at 30 years, and the biomedical assessment at 46 years of age. Study participants gave written informed consent. This manuscript adheres to the guidelines for Strengthening the Reporting of Observational studies in Epidemiology.^6^

Homelessness was reported at 30 years of age. It was defined as ever having to move out of a residence and having nowhere permanent to live since 16 years of age. Respondents answering positively were then asked where they stayed with the options of rough sleeping, a hostel or night shelter for the homeless, squatting (unlawfully staying in an uninhabited building or settling on a piece of land), bed and breakfasts/hotels, sofa surfing (staying with a friend or relative) and ‘other’ places, with multiple responses permitted. Information was also captured on early life confounding factors including socioeconomic disadvantage (leaving school <16 years old, highest educational achievement, unemployment), poor mental health (score of ≥7 on the Rutter Malaise Inventory,^7^ having specialist treatment for a psychiatric problem since age 16), smoking status, alcohol problems (defined as a score ≥2 on the cutting down, being annoyed by criticism, feeling guilty, and eye-openers (CAGE) questionnaire)^8^ and illicit drug use.

The biomedical examination included blood pressure assessment using the automated Omron HEM-907 device, height and weight measurement and drawing of non-fasting blood samples for analysis of lipids, C-reactive protein (CRP), insulin-like growth factor 1 and glycated haemoglobin (HbA1c). We adjusted risk factors for medication use by adding constant of +18% to the original value of triglycerides and −5% to high-density lipoprotein (HDL) cholesterol values in those on lipid-lowering drugs; +10 mm Hg to systolic and diastolic blood pressure in those treated for high blood pressure; and, +11 mmol/mol to HbA1c in those taking oral medication for type 2 diabetes.^9^ The adding of a constant value was chosen as it has been found to provide more power and prevents shrinkage of estimates compared with other approaches such as adding receipt of treatment as a binary covariate.^10^ Next, the Framingham model was used to calculate the 10-year risk of developing coronary heart disease.^11^

We compared the characteristics of participants with and without complete data. Multivariable linear regression was used to summarise the association between exposure to homelessness and biomedical risk factors. In preliminary analyses, there was no difference in results in men and women, so data were pooled and sex-adjusted. Exposure to homelessness was modelled as a binary variable. The primary analysis was the testing of associations between any exposure to homelessness and outcomes. Exploratory analyses were then conducted examining the place where participants stayed while homeless. The reference categories were not having been exposed to homelessness or that place of residence. Unstandardised regression coefficients were initially adjusted for age and sex, then socioeconomic disadvantage, poor mental health, smoking status, having an alcohol problem and lifetime illicit drug use. In analyses into the types of homeless experience, as multiple responses were permitted estimates were also mutually adjusted for all types of experience. All analyses were performed using Stata, V.16.1.

## Results

Between 2016 and 2018, 51.8% (n=8581) of the original cohort participated in a nurse-administered biomedical examination. Study members who did not take part in this examination were less likely at 30 years of age to have reported: being homeless (5.8% vs 7.1%), unemployed (2.2% vs 5.1%), leaving school before the minimum age of 16 (1.8% vs 3.3%), psychological distress (11.5% vs 15.1%), smoking (26.8% vs 34.0%), illicit drug use (51.2% vs 54.7%) and having a higher education qualification (23.6% vs 14.2%) (two-sided p values ≤0.01 for all differences).

Of those who took part in the biomedical survey, 429 (5.8%) had been homeless. Sofa surfing was the most common type of homeless experience (4.3%), followed by staying in a hostel (1%), another place (0.9%), bed and breakfast (0.6%), sleeping rough (0.4%) and, finally, a squat (0.2%). Study members who had been homeless were around four times more likely to have left school before 16 years of age (6.2% vs 1.5%), be unemployed (7.2% vs 1.9%), report psychological distress (22.7% vs 10.8%), been treated for a psychiatric problem (47.1% vs 23.5%), smoked (52.9% vs 25.1%), had an alcohol problem (20.3% vs 11.4%) and used illicit drugs (75.1% vs 53.4%).

The age-adjusted and sex-adjusted estimates for body mass index (BMI) and CRP were slightly raised, and HDL cholesterol lower, in those who had been homeless compared with those who had not (table 1). After adjustment for covariates assessed in early adulthood associations were attenuated. The relation with HDL was reduced to −0.09 (95% CI −0.13 to –0.03) and CRP to 0.14 (95% CI −0.03 to 0.31). Next, we examined the influence of where study members stayed when homeless. Staying in a bed and breakfast was associated with a higher BMI and CRP levels, sofa surfing with lower levels of HDL cholesterol and squatting with a *lower* BMI. Rough sleeping was associated with a higher 10-year risk of developing coronary heart disease. After adjusting for early-life characteristics, the association for squatting and BMI was lost and others attenuated (see online supplemental tables 1–6). Owing to missing data the sample size varied across analysis; however, sensitivity analyses in the non-missing sample did not alter conclusions (online supplemental table 7).

10.1136/jech-2021-217457.supp1 Supplementary data

**Table 1:** Association of homelessness in early adulthood with biomedical risk factors in middle age

Biomarker	Homeless, mean (SD)		Analytical sample size	Age-adjusted and sex-adjusted		Analytical sample size	Adjusted*	
	No	Yes		Coefficient	95% CI		Coefficient	95% CI
Body mass index	28.38 (5.46)	28.96 (6.06)	6431	0.61	0.03 to 1.19	6185	0.51	−0.09 to 1.10
Systolic blood pressure, mm Hg	125.21 (15.87)	123.70 (14.91)	6528	−0.68	−2.25 to 0.89	6280	−0.88	−2.49 to 0.74
Diastolic blood pressure, mm Hg	77.90 (11.57)	77.11 (11.92)	6528	−0.41	−1.59 to 0.78	6280	−0.67	−1.90 to 0.55
Total cholesterol, mmol/L	5.40 (1.00)	5.33 (1.04)	5293	−0.03	−0.15 to 0.08	5084	−0.09	−0.21 to 0.03
HDL cholesterol, mmol/L	1.53 (0.45)	1.44 (0.38)	5283	−0.11	−0.16 to –0.06	5074	−0.08	−0.13 to 0.03
Triglycerides, mmol/L	1.90 (1.51)	1.92 (1.40)	2977	0.14	−0.08 to 0.36	2865	−0.03	−0.26 to 0.20
Glycated haemoglobin, mmol/mol	37.05 (9.26)	37.17 (7.14)	5239	0.27	−0.82 to 1.35	5033	−0.41	−1.53 to 0.72
Framingham risk score†	5.36 (4.45)	5.63 (5.03)	5184	0.02	−0.03 to 0.08	4978	−0.03	−0.08 to 0.02
C reactive protein, log units	0.16 (1.08)	0.39 (1.08)	2947	0.22	0.06 to 0.39	2836	0.14	−0.03 to 0.31
Insulin-like growth factor 1, nmol/L	18.39 (5.00)	17.88 (4.88)	2972	−0.43	−1.19 to 0.34	2861	−0.08	−0.87 to 0.71

*Adjusted for age and sex plus socioeconomic disadvantage (leaving school aged <16 years old, highest educational achievement, being unemployed), psychiatric morbidity (Malaise score ≥7) and having seen doctor for psychiatric problem, smoking status, alcohol problems (CAGE score ≥2) and lifetime illicit drug use.

†Age, sex and current smoking not adjusted in the analyses of the Framingham risk score as they are used in its calculation.

HDL, high-density lipoprotein.;

## Discussion

Our main finding was that homelessness in early adult life was largely unrelated to a range of biomedical risk factors for mortality by middle age. After accounting for early-life characteristics inconsistent associations were found for staying in a bed and breakfast, squatting and rough sleeping. The large number of analyses, and attenuation observed following adjustment, means the few associations we found could reflect the play of chance or be due to residual confounding. These results contrast with those from case–control studies which have consistently found elevated biomedical risks in people who are homeless at the time of assessment.^12^ That this consistent pattern of elevated risk was not apparent suggests risks may have resolved to normative levels as participants had been rehoused. The household sampling used in the present cohort is likely to have resulted in a less extreme homeless experience than previous studies with rough sleepers and those attending homeless shelters.^13^ That sofa surfing was the most common type of experience that supports this hypothesis. We did, however, replicate associations between homelessness and greater socioeconomic disadvantage,^12^ mental health problems^14^ and substance use,^15^ but these differences were not physiologically expressed.

The main strength of this study is it is the first to assess the association between an array of biomedical risk factors according to the type of homelessness experienced. The lack of investigation into the less visible forms of homelessness is important as sofa surfing and bed and breakfast use account for over half of all homeless in England.^16^ The detailed assessments collected in the cohort of illicit drug use, alcohol problems, smoking and mental health problems, allowed us to examine a wide range of potential confounds that were self-reported, rather than assumed from death records, although some factors (eg, diagnoses of psychiatric disorders) were unavailable.

Our work is of course not without its limitations. As we used household sampling, tracking may have failed to find those who had become homeless by the biomedical assessment. Equally, as exposure to homelessness was only assessed at one time point transitions into homeless and those that occurred before 16 years of age would also have been missed. Some characteristics recorded in early adulthood could be characterised as mediators leading to concerns of overcontrol. The lack of association with outcomes in the minimally adjusted models suggests this will have had little effect on results. Approximately half of the original cohort members took part in the biomedical survey, with participation lower in those who had been homeless. It is not clear whether this would have biased estimates. If those lost were homeless and had a more adverse biomedical risk profile, this would have biased estimated towards the null. However, others have found that provided there was still normal variation in exposure and outcomes in the remaining members, attrition does not always affect findings.^17^

In conclusion, we found a history of homelessness in early adult life was unrelated to a range of established risk factors for mortality by middle age. We observed novel associations for bed and breakfast use, squatting and rough sleeping with some biomedical risk factors. These associations require replication, ideally with alternative cohorts and study designs.

**What is already known on this subject**

- Homelessness has been shown to be associated with an increased risk of premature mortality.
- Few studies have examined associations with risk factors in people who have been exposed to homelessness in early life.

**What this study adds**

- Exposure to homelessness was associated with greater socioeconomic disadvantage, mental health problems and substance use.
- Homelessness was not consistently related to any of the biomarkers.
