# Supplementary material for: Homelessness in early adulthood and biomedical risk factors by middle-age: the 1970 British Cohort Study
Source: J Epidemiol Community Health. 2021 Sep 28;76(4):382–4. doi: 10.1136/jech-2021-217457 (PMC8917972; doi:10.1136/jech-2021-217457)
Supplement: Supplementary data [file jech-2021-217457supp001.pdf]

Table S1. Association of having slept rough in early adulthood with biomedical risk factors in middle age

Biomarker	Analytical sample size	Minimal adjustment [a]		Analytical sample size	Multivariable adjustment [b]	
		Coefficient	95% CI		Coefficient	95% CI
Body mass index	5,937	-1.75	-4.17, 0.66	5,708	-1.79	-4.20, 0.60
Systolic blood pressure, mm Hg	6,030	-3.73	-10.36, 2.90	5,798	-3.55	-10.17, 3.06
Diastolic blood pressure, mm Hg	6,030	-3.93	-8.93, 1.08	5,798	-3.76	-8.78, 1.25
Total cholesterol, mmol/L	4,914	0.06	-0.44, 0.57	4,716	0.02	-0.48, 0.52
HDL cholesterol, mmol/L	4,904	0.03	-0.18, 0.24	4,706	0.04	-0.17, 0.26
Triglycerides, mmol/L	2,776	0.15	-0.74, 1.05	2,670	0.11	-0.79, 1.01
Glycated hemoglobin, mmol/mol	4,860	1.04	-3.46, 5.54	4,665	0.78	-3.74, 5.30
Framingham risk score [c]	4,814	0.04	0.01, 0.06	4,619	0.03	0.01, 0.05
C-reactive protein, log units	2,747	0.12	-0.55, 0.79	2,642	0.08	-0.58, 0.74
Insulin-like growth factor 1, nmol/L	2,771	-0.31	-3.38, 2.76	2,666	-0.28	-3.34, 2.79

Abbreviations: CI, confidence interval; HDL, high-density lipoprotein; SD, standard deviation.

[a] Adjusted for age, sex, other types of homeless experience (staying in a hostel, bed and breakfast, sofa surfing, squatting, other place)
[b] Adjusted for age and sex plus socioeconomic disadvantage (leaving school aged <16 years old, highest educational achievement, being unemployed), psychiatric morbidity (Malaise score ≥7) and having seen doctor for psychiatric problem, smoking status, alcohol problems (CAGE score ≥2), lifetime illicit drug use and other types of homeless experience.
[c] Age, sex and current smoking not adjusted in the analyses of the Framingham risk score as they are used in its calculation.

Table S2. Association of having stayed in a homeless shelter in early adulthood with biomedical risk factors in middle age

Biomarker	Analytical sample size	Minimal adjustment [a]		Analytical sample size	Multivariable adjustment [b]	
		Coefficient	95% CI		Coefficient	95% CI
Body mass index	5,937	0.15	-0.24, 0.54	5,708	0.10	-0.29, 0.49
Systolic blood pressure, mm Hg	6,030	0.83	-0.22, 1.89	5,798	0.83	-0.24, 1.90
Diastolic blood pressure, mm Hg	6,030	0.65	-0.15, 1.45	5,798	0.65	-0.15, 1.46
Total cholesterol, mmol/L	4,914	0.04	-0.04, 0.11	4,716	0.04	-0.04, 0.12
HDL cholesterol, mmol/L	4,904	-0.01	-0.05, 0.02	4,706	-0.01	-0.04, 0.02
Triglycerides, mmol/L	2,776	0.06	-0.09, 0.21	2,670	0.05	-0.10, 0.20
Glycated hemoglobin, mmol/mol	4,860	0.08	-0.61, 0.77	4,665	-0.09	-0.80, 0.61
Framingham risk score [c]	4,814	-0.01	-0.01, 0.01	4,619	0.00	-0.01, 0.01
C-reactive protein, log units	2,747	0.09	-0.02, 0.21	2,642	0.09	-0.02, 0.20
Insulin-like growth factor 1, nmol/L	2,771	0.11	-0.40, 0.62	2,666	0.13	-0.39, 0.65

Abbreviations: CI, confidence interval; HDL, high-density lipoprotein; SD, standard deviation.

[a] Adjusted for age, sex, other types of homeless experience (staying in a rough sleeping, bed and breakfast, sofa surfing, squatting, other place)
[b] Adjusted for age and sex plus socioeconomic disadvantage (leaving school aged <16 years old, highest educational achievement, being unemployed), psychiatric morbidity (Malaise score ≥7) and having seen doctor for psychiatric problem, smoking status, alcohol problems (CAGE score ≥2), lifetime illicit drug use and other types of homeless experience.
[c] Age, sex and current smoking not adjusted in the analyses of the Framingham risk score as they are used in its calculation.

Table S3. Association of having stayed in a bed and breakfast in early adulthood with biomedical risk factors in middle age

Biomarker	Analytical sample size	Minimal adjustment [a]		Analytical sample size	Multivariable adjustment [b]	
		Coefficient	95% CI		Coefficient	95% CI
Body mass index	5,937	0.58	0.13, 1.03	5,708	0.59	0.13, 1.05
Systolic blood pressure, mm Hg	6,030	0.36	-0.93, 1.55	5,798	0.12	-1.12, 1.36
Diastolic blood pressure, mm Hg	6,030	0.63	-0.27, 1.53	5,798	0.39	-0.55, 1.33
Total cholesterol, mmol/L	4,914	-0.06	-0.15, 0.02	4,716	-0.06	-0.15, 0.03
HDL cholesterol, mmol/L	4,904	-0.03	-0.06, 0.01	4,706	-0.02	-0.05, 0.02
Triglycerides, mmol/L	2,776	0.07	-0.08, 0.23	2,670	0.04	-0.12, 0.20
Glycated hemoglobin, mmol/mol	4,860	0.36	-0.43, 1.15	4,665	0.26	-0.55, 1.08
Framingham risk score [c]	4,814	-0.01	-0.01, 0.01	4,619	0.00	-0.01, 0.01
C-reactive protein, log units	2,747	0.18	0.06, 0.29	2,642	0.16	0.04, 0.29
Insulin-like growth factor 1, nmol/L	2,771	-0.06	-0.59, 0.47	2,666	0.02	-0.52, 0.56

Abbreviations: CI, confidence interval; HDL, high-density lipoprotein; SD, standard deviation.

[a] Adjusted for age, sex, other types of homeless experience (rough sleeping, staying in a hostel, sofa surfing, squatting, other place)
[b] Adjusted for age and sex plus socioeconomic disadvantage (leaving school aged <16 years old, highest educational achievement, being unemployed), psychiatric morbidity (Malaise score ≥7) and having seen doctor for psychiatric problem, smoking status, alcohol problems (CAGE score ≥2), lifetime illicit drug use and other types of homeless experience.
[c] Age, sex and current smoking not adjusted in the analyses of the Framingham risk score as they are used in its calculation.

Table S4. Association of having sofa surfed in early adulthood with biomedical risk factors in middle age

Biomarker	Analytical sample size	Minimal adjustment [a]		Analytical sample size	Multivariable adjustment [b]	
		Coefficient	95% CI		Coefficient	95% CI
Body mass index	5,937	0.45	-0.31, 1.20	5,708	0.44	-0.32, 1.19
Systolic blood pressure, mm Hg	6,030	-1.33	-3.38, 0.71	5,798	-1.36	-3.43, 0.71
Diastolic blood pressure, mm Hg	6,030	-1.02	-2.56, 0.52	5,798	-1.13	-2.70, 0.44
Total cholesterol, mmol/L	4,914	-0.01	-0.17, 0.14	4,716	-0.07	-0.23, 0.08
HDL cholesterol, mmol/L	4,904	-0.09	-0.15, -0.02	4,706	-0.07	-0.13, 0.01
Triglycerides, mmol/L	2,776	0.10	-0.22, 0.41	2,670	-0.06	-0.38, 0.25
Glycated hemoglobin, mmol/mol	4,860	0.36	-1.03, 1.76	4,665	-0.08	-1.49, 1.33
Framingham risk score [c]	4,814	0.00	-0.01, 0.01	4,619	0.01	-0.01, 0.01
C-reactive protein, log units	2,747	0.05	-0.18, 0.28	2,642	-0.03	-0.26, 0.21
Insulin-like growth factor 1, nmol/L	2,771	-0.63	-1.69, 0.43	2,666	-0.29	-1.36, 0.78

Abbreviations: CI, confidence interval; HDL, high-density lipoprotein; SD, standard deviation.

[a] Adjusted for age, sex, other types of homeless experience (rough sleeping, staying in a hostel, bed and breakfast, squatting, other place)
[b] Adjusted for age and sex plus socioeconomic disadvantage (leaving school aged <16 years old, highest educational achievement, being unemployed), psychiatric morbidity (Malaise score ≥7) and having seen doctor for psychiatric problem, smoking status, alcohol problems (CAGE score ≥2), lifetime illicit drug use and other types of homeless experience.
[c] Age, sex and current smoking not adjusted in the analyses of the Framingham risk score as they are used in its calculation.

Table S5. Association of having squatted in early adulthood with biomedical risk factors in middle age

Biomarker	Analytical sample size	Minimal adjustment [a]		Analytical sample size	Multivariable adjustment [b]	
		Coefficient	95% CI		Coefficient	95% CI
Body mass index	5,937	-1.73	-3.13, -0.32	5,708	-1.69	-3.08, -0.29
Systolic blood pressure, mm Hg	6,030	-2.83	-6.68, 1.03	5,798	-2.78	-6.62, 1.07
Diastolic blood pressure, mm Hg	6,030	0.03	-2.88, 2.94	5,798	-0.02	-2.94, 2.90
Total cholesterol, mmol/L	4,914	-0.19	-0.52, 0.14	4,716	-0.20	-0.53, 0.13
HDL cholesterol, mmol/L	4,904	-0.04	-0.18, 0.10	4,706	-0.04	-0.17, 0.10
Triglycerides, mmol/L	2,776	-0.35	-1.00, 0.30	2,670	-0.36	-1.02, 0.28
Glycated hemoglobin, mmol/mol	4,860	-0.31	-3.26, 2.64	4,665	-0.52	-3.47, 2.43
Framingham risk score [c]	4,814	0.00	-0.01, 0.01	4,619	-0.01	-0.02, 0.00
C-reactive protein, log units	2,747	0.06	-0.43, 0.54	2,642	0.11	-0.37, 0.59
Insulin-like growth factor 1, nmol/L	2,771	2.09	-0.13, 4.33	2,666	2.15	-0.08, 4.37

Abbreviations: CI, confidence interval; HDL, high-density lipoprotein; SD, standard deviation.

[a] Adjusted for age, sex, other types of homeless experience (rough sleeping, staying in a hostel, bed and breakfast, sofa surfing, other place)
[b] Adjusted for age and sex plus socioeconomic disadvantage (leaving school aged <16 years old, highest educational achievement, being unemployed), psychiatric morbidity (Malaise score ≥7) and having seen doctor for psychiatric problem, smoking status, alcohol problems (CAGE score ≥2), lifetime illicit drug use and other types of homeless experience.
[c] Age, sex and current smoking not adjusted in the analyses of the Framingham risk score as they are used in its calculation.

Table S6. Association of having stayed in other places in early adulthood with biomedical risk factors in middle age

Biomarker	Analytical sample size	Minimal adjustment [a]		Analytical sample size	Multivariable adjustment [b]	
		Coefficient	95% CI		Coefficient	95% CI
Body mass index	5,937	1.32	-0.15, 2.79	5,708	1.29	-0.20, 2.77
Systolic blood pressure, mm Hg	6,030	0.28	-3.69, 4.24	5,798	0.50	-3.53, 4.52
Diastolic blood pressure, mm Hg	6,030	-1.16	-4.16, 1.83	5,798	-1.18	-4.23, 1.88
Total cholesterol, mmol/L	4,914	-0.17	-0.46, 0.12	4,716	-0.24	-0.53, 0.05
HDL cholesterol, mmol/L	4,904	-0.10	-0.22, 0.02	4,706	-0.10	-0.22, 0.02
Triglycerides, mmol/L	2,776	0.15	-0.34, 0.64	2,670	0.06	-0.44, 0.54
Glycated hemoglobin, mmol/mol	4,860	1.25	-1.33, 3.82	4,665	0.94	-1.67, 3.55
Framingham risk score [c]	4,814	0.01	-0.01, 0.01	4,619	0.00	-0.02, 0.01
C-reactive protein, log units	2,747	0.01	-0.36, 0.38	2,642	-0.02	-0.39, 0.35
Insulin-like growth factor 1, nmol/L	2,771	-0.50	-2.18, 1.19	2,666	-0.27	-1.95, 1.42

Abbreviations: CI, confidence interval; HDL, high-density lipoprotein; SD, standard deviation.

[a] Adjusted for age, sex, other types of homeless experience (rough sleeping, staying in a hostel, bed and breakfast, sofa surfing, squatting).
[b] Adjusted for age and sex plus socioeconomic disadvantage (leaving school aged <16 years old, highest educational achievement, being unemployed), psychiatric morbidity (Malaise score ≥7) and having seen doctor for psychiatric problem, smoking status, alcohol problems (CAGE score ≥2), lifetime illicit drug use and other types of homeless experience.
[c] Age, sex and current smoking not adjusted in the analyses of the Framingham risk score as they are used in its calculation.

Table S7. Association of having been homelessness in early adulthood with biomedical risk factors in the sample with no missing data (n = 2662)

Biomarker	Homeless, Mean (SD)		Age- and sex-adjusted		Adjusted [a]	
	No	Yes	Coefficient	95% CI	Coefficient	95% CI
Body mass index	28.52 (5.46)	28.88 (6.09)	0.43	-0.46, 1.32	0.33	-0.57, 1.24
Systolic blood pressure, mm Hg	125.08 (15.55)	122.13 (15.02)	-1.80	-4.19, 0.58	-1.80	-4.24, 0.63
Diastolic blood pressure, mm Hg	77.66 (11.47)	75.62 (12.58)	-1.51	-3.34, 0.32	-1.41	-3.28, 0.46
Total cholesterol, mmol/L	5.36 (0.97)	5.27 (1.01)	-0.06	-0.22, 0.09	-0.09	-0.26, 0.06
HDL cholesterol, mmol/L	1.52 (0.44)	1.45 (0.38)	-0.09	-0.15, -0.02	-0.06	-0.13, 0.01
Triglycerides, mmol/L	1.86 (1.24)	1.90 (1.40)	0.14	-0.05, 0.33	0.01	-0.19, 0.19
Glycated hemoglobin, mmol/mol	36.68 (7.89)	36.61 (5.24)	0.11	-1.14, 1.37	0.33	-1.59, 0.95
Framingham risk score [b]	5.56 (4.60)	5.45 (5.35)	-0.01	-0.01, 0.01	-0.01	-0.01, 0.01
C-reactive protein, log units	0.15 (1.06)	0.38 (1.09)	0.23	0.05, 0.39	0.16	-0.01, 0.34
Insulin-like growth factor 1, nmol/L	18.45 (4.98)	17.86 (4.93)	-0.52	-1.33, 0.28	-0.16	-0.98, 0.66

Abbreviations: CI, confidence interval; HDL, high-density lipoprotein; SD, standard deviation.

[a] Adjusted for age and sex plus socioeconomic disadvantage (leaving school aged <16 years old, highest educational achievement, being unemployed), psychiatric morbidity (Malaise score ≥7) and having seen doctor for psychiatric problem, smoking status, alcohol problems (CAGE score ≥2) and lifetime illicit drug use.

[b] Age, sex and current smoking not adjusted in the analyses of the Framingham risk score as they are used in its calculation.
